# Expression of the leukemic prognostic marker CD7 is linked to epigenetic modifications in chronic myeloid leukemia

**DOI:** 10.1186/1476-4598-9-41

**Published:** 2010-02-22

**Authors:** Sally L Rogers, Yun Zhao, Xiaoyan Jiang, Connie J Eaves, Dixie L Mager, Arefeh Rouhi

**Affiliations:** 1 Terry Fox Laboratory, British Columbia Cancer Agency, 675 West 10th Avenue, Vancouver, British Columbia V5Z 1L3, Canada; 2 Department of Medical Genetics, University of British Columbia, 2350 Health Sciences Mall, Vancouver, British Columbia V6T 1Z3, Canada; 3 Current address: Institute of Experimental Cancer Research, Comprehensive Cancer Centre, University Hospital Ulm, Albert-Einstein-Allee 11, 89081 Ulm, Germany

## Abstract

**Background:**

Expression levels of the cell surface glycoprotein, CD7, and the serine protease, elastase 2 (ELA2), in the leukemic cells of patients with chronic myeloid leukemia (CML) have been associated with clinical outcome. However, little is known about the mechanisms that underlie the variable expression of these genes in the leukemic cells.

**Results:**

To address this question, we compared the level of their expression with the DNA methylation and histone acetylation status of 5' sequences of both genes in leukemic cell lines and primitive (lin^-^CD34^+^) leukemic cells from chronic phase CML patients. DNA methylation of the *ELA2 *gene promoter did not correlate with its expression pattern in lin^-^CD34^+ ^cells from chronic phase CML patient samples even though there was clear differential DNA methylation of this locus in *ELA2*-expressing and non-expressing cell lines. In contrast, we found a strong relation between CD7 expression and transcription-permissive chromatin modifications, both at the level of DNA methylation and histone acetylation with evidence of hypomethylation of the *CD7 *promoter region in the lin^-^CD34^+ ^cells from CML patients with high CD7 expression.

**Conclusion:**

These findings indicate a link between epigenetic modifications and CD7 expression in primitive CML cells.

## Background

A feature of chronic myeloid leukaemia (CML) is the Philadelphia chromosome (Ph). This abnormal chromosome results from a reciprocal translocation between chromosome 9 and 22 that gives rise to the *BCR-ABL *fusion gene that is now the accepted defining hallmark of the disease [1-3]. CML is typically diagnosed in an initial chronic phase (CP) which is characterized by the formation of a multi-lineage clone of Ph^+^/*BCR-ABL*^+ ^leukemic cells that typically dominates the entire hematopoietic system by the time of diagnosis. This clone includes a selectively enlarged compartment of myeloid progenitors that produce an elevated number of normally differentiated granulocytes [4]. Targeted therapy of CML with imatinib mesylate (IM) or other inhibitors of the BCR-ABL oncoprotein is the current therapy of choice for patients with CP disease, although IM does not always produce durable remissions [5,6]. The most common cause of a poor response outcome is the appearance of IM-resistant cells. This resistance might be BCR-ABL-dependent, such as mutations in the kinase domain of BCR-ABLand genomic amplification of the *BCR-ABL *locus or BCR-ABL-independent mechanisms such as constitutive activation of downstream pathways [7-9]. Other genetic causes of disease heterogeneity as well as the inherent resistance of the leukemic progenitor/stem cells to IM may be additional contributing factors [2,10,11]. Identification and characterization of epigenetic changes that control the properties of CML cells, especially those of CML stem/progenitor (lin^-^CD34^+^) cells may be useful for the design of suitable therapies for IM-refractory CML [12].

As with other cancers, various markers divide CP CML into different subclasses that are associated with different patient survival probabilities. *CD7 *and elastase 2 (*ELA2) *are two such genes that show differential expression patterns in CML cells [13]. Various studies indicate expression of *CD7 *to be upregulated in CML cells (and various other leukaemias and lymphomas) where it has been associated with poor survival [14,15]. *ELA2 *is clustered with two other serine protease gene family members, azurocidin 1 and proteinase 3 (*PRTN3*, also known as Myeloblastin) genes, at chromosome 19pter [16]. These three genes are expressed co-ordinately and their products packaged together into azurophil granules during neutrophil differentiation. High expression of these three genes in CML is associated with a favourable prognosis [13].

*CD7 *is located on chromosome 17 and encodes a cell surface glycoprotein member of the immunoglobulin superfamily. The protein is found on thymocytes and mature NK and T-cells [17,18]. It plays an essential role in T-cell interactions and also in T-cell/B-cell interactions during early lymphopoeisis. *ELA2 *is one of six structurally similar human elastase genes, *ELA1, 2, 2A, 2B, 3A *and *3B*. Elastases form a subfamily of serine proteases that hydrolyze many proteins in addition to elastin. ELA2 hydrolyzes proteins within specialized neutrophil lysosomes, called azurophil granules, as well as proteins of the extracellular matrix following the protein's release from activated neutrophils. In addition, ELA2 may play a role in degenerative and inflammatory diseases by its proteolysis of collagen-IV and elastin of the extracellular matrix. This protease degrades the outer membrane protein A (OmpA) of E. coli as well as the virulence factors of such bacteria as Shigella, Salmonella and Yersinia [19]. Mutations in the *ELA2 *gene are associated with cyclic neutropenia and severe congenital neutropenia (SCN) [20].

The role of epigenetic mechanisms in the transcriptional control of *CD7 *and *ELA2 *is unknown. In this study we have examined the transcription pattern of these two genes in normal adult human bone marrow and primary CML cells. We specifically compared the degree of 5' region DNA methylation with *CD7 *and *ELA2 *transcript levels and assessed levels of histone acetylation at the promoter regions of *CD7 *and *ELA2 *in expressing and non-expressing cell lines.

## Materials and methods

### Cells

The human cell line THP-1 (monocytic leukemia) was cultured in RPMI plus 10% fetal calf serum (FCS), 10 mM Hepes, 2 mM glutamine and penicillin/streptomycin (pen/strep). The human cell lines ALL-SIL (T-cell leukemia) and RPM1 (T-ALL) were cultured in RPMI plus 10% FCS, 1% sodium pyruvate, 1% glutamine and pen/strep. Normal adult human bone marrow cells and CP CML patient samples were obtained with informed consent according to protocols approved by the Research Ethics Board of the University of British Columbia. Heparin-treated blood and leukapheresis cells were obtained from 12 chronic phase CML patients with elevated white blood cell counts (Table 1) at the time of their initial diagnosis. None of the 12 patients had been treated with imatinib, and all lacked any clinical evidence of accelerated disease when the samples were taken.

**Table 1:** Clinical data from 12 CML patients studied

Patient no.	Age at diagnosis	Sex	WBC at diagnosis (× 10^3^/mL)	Disease status at diagnosis	Disease progression^a^
1	40	M	296	CP	AP
2	54	M	66	CP	AP
3	66	F	492	CP	AP
4	34	M	494	CP	CP
5	56	F	97	CP	CP
6	44	M	156	CP	CP
7	25	M	164	CP	CP
8	60	M	81	CP	CP
9	47	F	190	CP	AP
10	54	M	166	CP	BC
11	60	M	15	CP	CP
12	46	F	161	CP	CP

^**a**^CP = chronic phase, AP = accelerated phase and BC = blast crisis

Light density cells were first isolated by centrifugation on ficoll-hypaque and then cells expressing the following lineage (lin) markers: CD2, CD3, CD14, CD16, CD19, CD24, CD56, CD66b and glycophorin A were removed immunomagnetically using a column (StemSep, StemCell Technologies, Vancouver, BC) as recommended by the manufacturer. Cells were then cryopreserved in DMSO plus FCS until required. After thawing, the lin^- ^cells were stained with allophycoerythrin (APC)-conjugated anti-human CD34 antibody (8G12, Becton Dickinson, San Jose, CA,) and propidium iodide (PI, Sigma Chemicals, St. Louis, MO). Viable (PI^-^) lin^-^CD34^+ ^cells (enriched in stem/progenitor cells) were then isolated using a FACSVantage flowcytometer (BD). Cells were further sorted, where indicated in the results, using fluorescein isothiocyanate (FITC)-conjugated anti-human CD7 antibody (M-T701, BD).

### DNA and cDNA preparation

DNA was prepared in the following manner. Cell lines were washed twice in PBS, then boiled in sterile water for 10 mins. Samples were incubated with proteinase K for two hours, followed by denaturation for 10 mins. DNA was isolated from CML and normal tissue samples using the Qiagen (Germantown, MD) DNA/RNA isolation kit, according to the manufacturer's recommendation. The PicoPure™ RNA extraction kit (Arturus, Mountainview, CA) was use to extract RNA, according to the manufacturer's protocol using DNaseI (Invitrogen, Carlsbad, CA) treatment during the procedure to minimize contamination with genomic DNA. RNA was reverse transcribed with SuperScriptIII (Invitrogen) to generate first strand cDNA,

### Bisulfite conversion and PCR amplification

Bisulfite conversion of DNA was performed using the EZ DNA methylation kit (Zymo Research, Orange, CA), according to the manufacturer's protocol, with minor modifications [21]. Treated DNA was cleaned and eluted to 15 μl following the manufacturer's instructions. PCR amplifications of two regions spanning the start of the *ELA2 *gene were performed on converted DNA using the following primers: ELA2BS1 for 5'-TGGGTTTTATTTGGAAGAGATTTAG-3' with ELA2BS1rev 5'-CCTCCAAACAAAATTCAAAATACAC-3', and ELA2BS2for 5'-GTGTATTTTGAATTTTGTTTGGAGG-3' with ELA2BS2rev 5'-CCTCAATCTCTTCTAATCTCC-3' with Platinum *Taq *(Invitrogen Life Technologies) under the following conditions: 94°C for 8 minutes, followed by 40-45 cycles of 94°C for 90 seconds, 55°C for 90 seconds, and 72°C for 45 seconds.

PCR amplification of the region spanning the start of the *CD7 *gene was performed on converted DNA using the following primers: CD7BS for 5'-TAGAGGATTAGGTAGGTTG-3' with CD7BSrev 5'-AACTCTTACCTTAAACAACC-3', with the same conditions as above apart from a 50°C annealing step. A final elongation step of 10 minutes was included in all reactions. PCR products were analyzed by gel electrophoresis and products purified with MiniElute (Qiagen).

### Sequencing

PCR products were cloned using the pGEM-T Easy kit (Promega, Madison, WI). Sequencing was performed by The McGill University and Genome Québec Innovation Centre Sequencing Platform. Only unique sequences (as determined by either unique CpG methylation pattern or unique non-conversion of non-CpG cytosines) are shown, and all sequences had a conversion rate of higher than 95%.

### Combined bisulfite and restriction analysis (CoBRA)

In addition to cloning and sequencing, PCR products were digested individually with the restriction enzymes *Hin*fI, *Mbo*I, *Taq*I, or *Rsa*I, which contain CpG dinucleotides in their recognition sites. If the CpG is methylated in the original sample, then the restriction enzyme site will be conserved during bisulfite treatment and the PCR product will be digested. All enzymes were purchased from New England Biolabs (Ipswich, MA), and digests were performed overnight following manufacturer's instructions. Control reactions were performed each time to ensure complete digestion, and uncut reactions were run on agarose gels with digests for comparison.

### Chromatin immunoprecipitation and quantitative PCR

**Figure 1:**
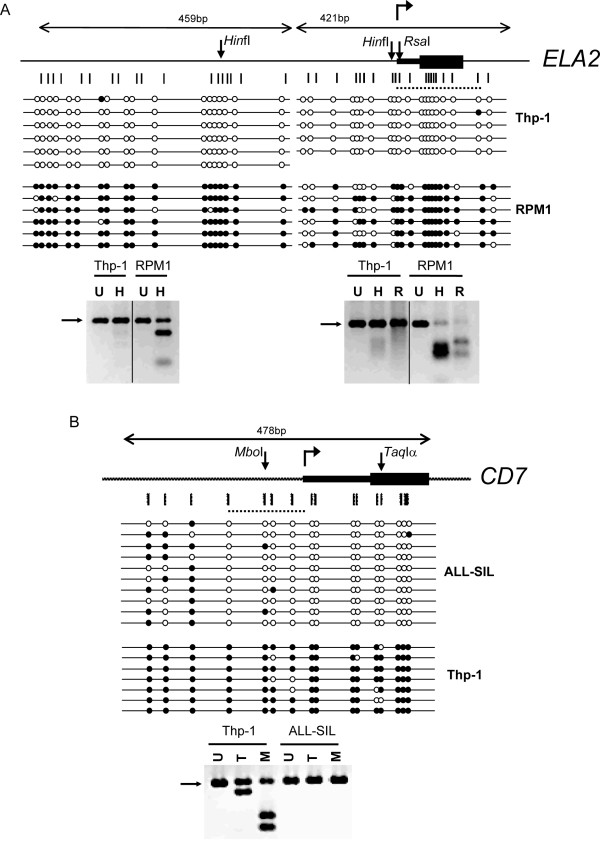
**Expression of *CD7 *and *ELA2 *is associated with DNA hypomethylation in leukemic cell lines**. Bisulfite sequencing and CoBRA for A) *ELA2*, on THP-1 (positive) and RPM1 (negative); and B) *CD7 *on THP-1 (negative) and ALL-SIL (positive). Cartoons show gene organization, with transcriptional start sites as bent arrow, UTR as narrow bars and translated regions as wide bars. Down arrows show location of enzyme sites used for CoBRA. Vertical bars show location of CpG dinucleotides. Dotted lines show location of region amplified after ChIP. Open and filled circles indicate unmethylated and methylated CpGs respectively. Bottom panels show CoBRA for each gene. Enzymes used are indicated as follows; U = uncut, H = *Hin*fI, R = *Rsa*, T = *Taq*Iα and M = *Mbo*I. Position of the uncut band is indicated by an arrow.

Chromatin immunoprecipitation (ChIP) assays were performed using a ChIP assay kit (Upstate Biotechnology, Billerica, MA) according to the manufacturer's instructions. The following antibodies were used to perform immunoprecipitations, polyclonal anti-acetyl-Histone H3 (Lysine-9) and polyclonal anti-acetyl-Histone H4 (multiple residues) (both Upstate Biotechnology). The DNA was purified via QIAquick PCR purification (Qiagen) and resuspended in 30 μl of de-ionized water. Quantitative PCR amplification of human *CD7 *and *ELA2 *sequences was performed on these samples using the following primers (location shown in figure 1): *CD7 *forward 5'-ACCTCCTCCCTGTGGAGATG-3', *CD7 *reverse 5'-AGAGCTCAGAGAGGGCTTCCT-3'; *ELA2 *forward, 5'-CAGCACAGGGCTATAAGAGG-3', *ELA2 *reverse 5'-GAGCAGCGGAGGTTGGAC-3'. The housekeeping gene *HPRT *was amplified as a positive control using forward primer 5'-CCCTCAGGCGAACCTCTCG-3' and reverse primer 5'-GGCTGCGGGTCGCCATAACG-3'. The neural filament gene *NFM *was amplified as a negative control using forward primer 5'-CATCTCGACGGCGCTGAAGG-3' and reverse primer 5'-GGTACTCGGCGATCTCTTCC-3'. 45 rounds of amplification with SYBR^® ^Green PCR Master Mix (Applied Biosystems, Foster City, CA) were performed. The default 7500 System SDS software version 1.2.10 (7500 RealTime PCR System, Applied Biosystems) cycle was used with the amplification performed at 60°C in a total volume of 25 μl. Dissociation curve analysis was performed at the end of each PCR to confirm the presence of a single and specific product.

### Absolute expression analysis using quantitative realtime PCR

Absolute quantification of *CD7, ELA2 *and *PRTN3 *cDNAs was performed according to Jiang et al. (2004) [22]. The following primers: *ELA2 *forward primer 5'-ACGACATCGTGATTCTCCAGCTCA-3', *ELA2 *reverse primer 5'-CGTTGAGCTCCTGCAGGAC-3', *CD7 *forward primer 5'-TAGACCCAGAGAGGCTCAG-3', *CD7 *reverse primer 5'-GGAGACTGCTGCACCTCTTGG-3'. The housekeeping gene *GAPDH *was amplified as an internal control using forward primer 5'-CCCATCACCATCTTCCAGGAG-3' and reverse primer 5'-CTTCTCCATGGTGGTGAAGACG-3'. 40 rounds of amplification with SYBR^® ^Green PCR Master Mix (Applied Biosystems) were performed. The default 7500 System SDS software version 1.2.10 (7500 RealTime PCR System, Applied Biosystems) cycle was used with amplification performed at 62°C in a total volume of 25 μl. Dissociation curve analysis was performed after the end of each PCR to confirm the presence of a single and specific product.

### Statistical analysis

P-values for the DNA methylation analysis were calculated using Fisher's exact test. The statistical significance of absolute quantities of *ELA2 *and *CD7 *cDNAs in normal and CML samples was calculated using the Mann-Whitney U-test.

## Results

### Transcription of CD7 and ELA2 is associated with DNA hypomethylation in leukemic cell lines

We first screened a panel of human leukemic cell lines for transcription of *CD7 *and *ELA2 *with RT-PCR (Additional File 1). THP-1 transcribes *ELA2 *but not *CD7*, ALL-SIL transcribes *CD7 *and RPM1 is negative for *ELA2*. We then assessed whether methylation patterns of the 5' CpG-rich regions of the *CD7 *and *ELA2 *genes are linked with their transcriptional activity. We identified a CpG rich region in the human *ELA2 *gene, from 606 nt upstream of the transcriptional start site to 247 nt downstream, including all of exon 1. This region does not contain any CpG islands according to the standard definition (> 50% G+C content, and observed/expected CpG ratio of > 0.6). However, it is CpG rich, with an observed to expected ratio of 0.5, compared to 0.1 across the human genome. It is also GC rich (58%), not only compared to the genome average of 41% GC, but also compared to the 48% GC average across chromosome 19 [23].

DNA was isolated from all three of these cell lines and treated with sodium bisulfite. Sodium bisulfite converts cytosine to uracil, but does not convert 5-methylcytosine, allowing the bases to be distinguished by PCR amplification. The 853-bp CpG-rich region of the *ELA2 *gene was PCR amplified from the treated DNA, cloned and sequenced (Figure 1A). CoBRA analysis was also performed on the PCR products, using two enzymes, as shown in Figure 1A, allowing analysis of three of the CpG dinucleotides. If the CpG was originally methylated, the restriction enzyme site will be retained in the treated DNA and the PCR product will be digested. The results of both the sequencing and the CoBRA show that the CpG rich region is significantly less methylated in the *ELA2*^+ ^THP-1 cell line than in the *ELA2*^- ^RPM1cell line, in which the majority of the CpGs are methylated. We also analyzed the DNA methylation status of *ELA2 *in the non-expressing (Additional File 1A) CML cell line, K562 (Additional File 2A), showing moderate to high methylation.

We also identified a CpG-rich region in the human *CD7 *gene, from 277 nt upstream of the transcriptional start site to 200 nt downstream of the transcriptional start site, and performed a similar analysis on this region (Figure 1B). This region also does not contain any CpG islands, but it is enriched for CpGs, with an observed to expected ratio of 0.3. It is also GC rich (69%) compared to the 45.5% GC average across chromosome 17 [24]. Bisulfite sequencing and CoBRA revealed that this region is significantly less methylated in the *CD7*^+ ^ALL-SIL cell line than in the *CD7*^- ^THP-1 cell line, in which the majority of CpGs are methylated. We also analyzed the DNA methylation status of *CD7 *in the low-expressing (Additional File 1B) CML cell line, K562 (Additional File 2B), which showed partial methylation.

### Expression of CD7 and ELA2 is associated with histone acetylation in leukemic cell lines

**Figure 2:**
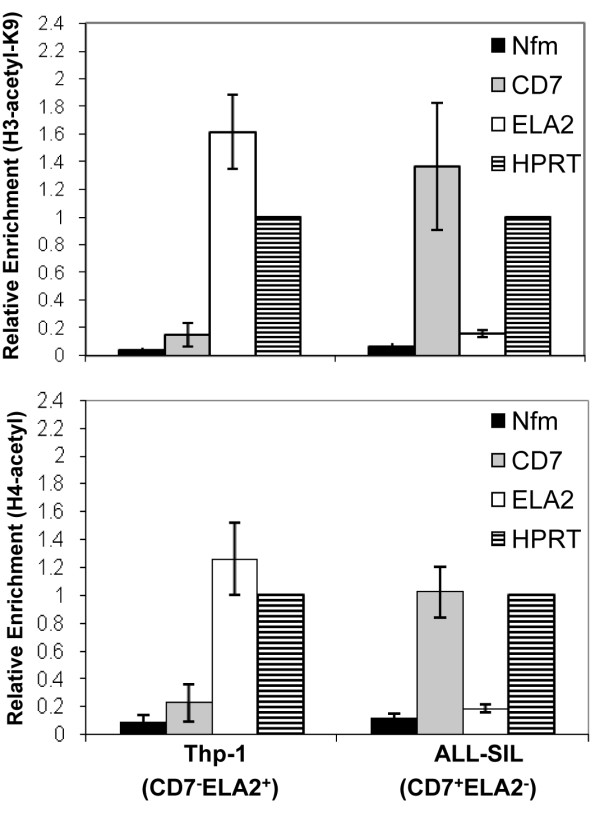
**Expression of *CD7 *and *ELA2 *is associated with histone acetylation in leukemic cell lines**. ChIP was performed on THP-1 (*CD7*^-^*ELA2*^+^) and ALL-SIL (*CD7*^+^*ELA2*^-^) cell lines with anti-acetyl-H3K9 and anti-pan-acetyl-H4 antibodies. Black bars indicate *NFM *(negative control); grey bars, *CD7*; white bars, *ELA2 *and striped bars, *HPRT *(positive control).

Studies suggest that CpG methylation is linked to histone deacetylation resulting in the formation of condensed, transcriptionally inactive chromatin. Acetylation of histones H3 and H4 is an epigenetic modification associated with an open chromatin structure and transcriptionally active genes [25]. The binding of acetylated histones to the *CD7 *and *ELA2 *CpG-rich regions in THP-1 (*CD7*^-^, *ELA2*^+^) and ALL-SIL (*CD7*^+^, *ELA2*^-^) cells was analyzed using ChIP. In these analyses, cross-linked chromatin was immunoprecipitated using anti-acetyl H3 Lys 9 or anti-acetyl H4 antibodies. As a negative control, we included a precipitation reaction containing no antibody, and the input fractions prior to immunoprecipitation were used as positive controls. After immunoprecipitation and reversal of the cross-links, enrichment of the *CD7 *and *ELA2 *CpG-rich fragments in each sample was measured by quantitative real-time PCR. The results are shown as the relative association of the tested gene regions with acetylated histones normalized to a positive control gene (*HPRT*), which is expressed in all the cell lines and set at a reference value of one (Figure 2). As an additional control, the same immunoprecipitate was also used to amplify a negative control DNA genomic region from the *NFM *gene, which is expressed exclusively in neurons (and therefore not in any of the cell lines tested). The results show that both *CD7 *and *ELA2 *CpG-rich regions are significantly associated with acetylated histones in the positive cell lines (ALL-SIL and THP-1, respectively) at levels as high as, or higher than, the positive control region of *HPRT*. In contrast, there is relatively little association of acetylated histones with the CpG-rich region of *CD7 *and *ELA2 *in the negative cell lines (THP-1 and ALL-SIL, respectively). These results indicate that acetylated histones H3 and H4 specifically bind to the CpG rich regions of *CD7 *and *ELA2 *in leukemic cell lines *in situ*.

### Range of ELA2 and CD7 transcript levels in CD34^+ ^cells from CML patients and normal controls

**Figure 3:**
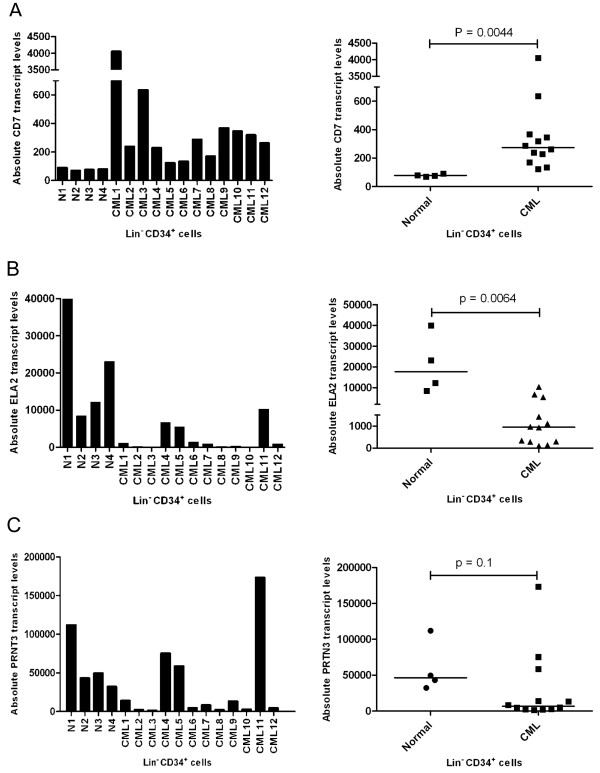
**Absolute quantification of transcripts of A) *CD7*, B) *ELA2 *and C) *PRTN3 *in normal and CML human samples**. Normal samples are all from bone marrow, CML 1-10 are from peripheral blood except for CML11 and 12 which are from bone marrow. Cells and cDNA were prepared as described in materials and methods. Median of transcript levels is indicated.

It has previously been reported that high CD7 and low ELA2 expression in CML cells is associated with a poor prognosis. In order to determine the range of expression in the samples of normal and CML lin^-^CD34^+ ^cells used in the present study (Table 1), we performed absolute quantification of transcript levels for both genes using real-time RT-PCR (Figure 3A and 3B). The amount of *CD7 *transcripts is relatively constant in the lin^-^CD34^+ ^cells from all four normal bone marrow samples, whereas the *CD7 *transcript levels in the corresponding subset of CML cells show marked variation among samples. Nevertheless, on average, the level of *CD7 *expression was significantly higher in the CML samples (P < 0.0001). *ELA2 *transcript levels were more variable within both the normal and CML groups although, on average, the levels in the CML samples were significantly lower (P = 0.0005). However, transcript levels of the co-regulated *PRTN3 *gene did not show a statistically significant difference between CML and normal groups (Figure 3C), due primarily to one CML sample (CML11) with very high levels of *PRTN3 *transcript.

### DNA hypomethylation correlates with increased CD7 expression on individual cells in CML

**Figure 4:**
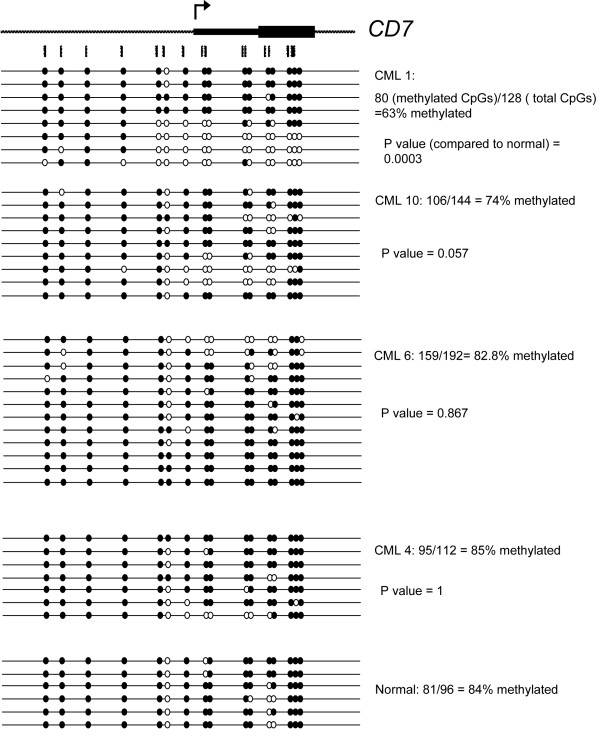
**DNA methylation analysis of *CD7 *in normal and CML samples**. CD7 is expressed highly in CML1 and intermediately in CML10 but CML4 and CML6 have close to normal levels of CD7 expression. Open and black fill circles indicate unmethylated and methylated CpG respectively.

To determine if the increased expression of *CD7 *in lin^-^CD34^+ ^CML cells is associated with hypomethylation of the *CD7 *promoter region, we isolated DNA from lin^-^CD34^+ ^cells from one normal (N1) and three CML (CML1, 4, 6 and 10) samples. These samples were chosen as the CML1, 10, 6 and 4 cells showed high, intermediate and low levels of CD7 expression, respectively (refer to Figure 3). Only one normal sample was chosen for sequencing as the expression level of CD7 is relatively constant in this group. Sequencing of bisulfite-treated DNA from these samples shows that the *CD7 *promoter region is significantly less methylated in the CML1 cells than in the normal sample (P = 0.0003, Figure 4). In contrast, the *CD7 *promoter regions in CML4 and CML6 have approximately the same percentage methylation as the normal sample. Interestingly, in CML10 (which has moderate expression of CD7), there is lower percentage methylation than in the normal sample approaching statistical significance. This is consistent with the results from Figure 1B suggesting that *CD7 *transcript levels are proportional to the extent of methylation of the *CD7 *promoter region. We also performed DNA methylation analysis of *CD7 *in bulk CD34^+ ^cells (containing both lin^- ^and lin^+ ^cells) from CML1 and CML3 and found similar results as for lin^-^CD34^+ ^CML cells (Additional File 3).

**Figure 5:**
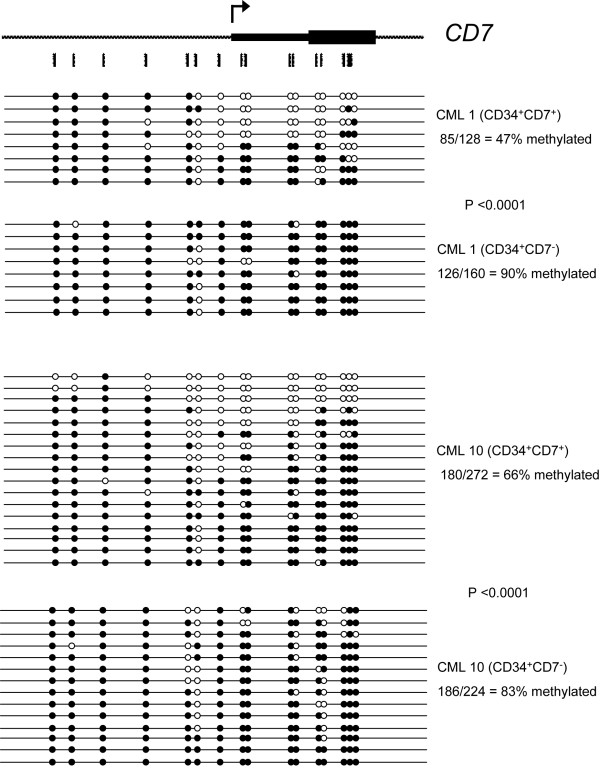
**Significantly less *CD7 *promoter methylation in CD7 expressing cells from CML1 and CML10**. CD34^+ ^cells of patient sample CML1 and CML10 were sorted into CD7^+ ^and CD7^- ^fractions and DNA methylation analysis was performed by sodium bisulfite sequencing. Open and black fill circles indicate unmethylated and methylated CpG respectively.

Detailed analysis of the methylation pattern of the sequences shown for CML1 in Figure 4 reveals that individual clones appear to be either unmethylated or methylated, as opposed to patchy methylation across all the CpG sites. One hypothesis for this pattern is that expression of CD7 on individual cells in the lin^-^CD34^+ ^population varies, and that those cells which are positive are demethylated at the *CD7 *promoter. To test this hypothesis, CD7^+ ^and CD7^- ^cells were isolated after staining CML1 and CML10 lin^-^CD34^+ ^cells with anti-CD7 and the methylation pattern of *CD7 *determined as before (Figure 5). Only 47% and 66% of the *CD7 *CpG sites tested are methylated in the CD7^+ ^cells of CML1 and CML10 respectively, compared to 90% (CML1) and 83% (CML10) in the CD7^- ^cells (P < 0.0001) suggesting that hypomethylation of the *CD7 *promoter is associated with higher expression of CD7 on individual cells in CML patients. Moreover, in both the cell lines and patient samples, it appears to be the region downstream from the fourth CpG site analysed into the transcribed portion of the gene that is demethylated. Interestingly, not all clones in the CD7^+ ^fraction show hypomethylation of the *CD7 *5'-region. This result cannot be due to contamination by CD7^- ^cells, because of the high purity of the sort. Rather, it is possible that in some of these CD7^+ ^cells expression is only promoted from one allele. We also performed DNA methylation analysis of *CD7 *in bulk CD34^+ ^cells (containing both lin^- ^and lin^+ ^cells) sorted for CD7 expression from CML3 and found similar results as for lin^-^CD34^+ ^CML cells (Additional File 4).

### DNA methylation and transcript levels of ELA2 in CML are not correlated

**Figure 6:**
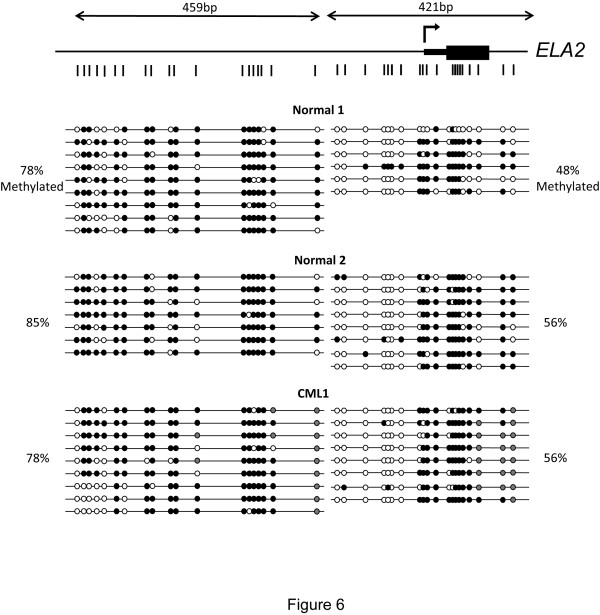
**DNA methylation analysis of *ELA2 *in normal and CML samples**. Open and black fill circles indicate unmethylated and methylated CpG respectively. Grey fill circles indicated CpG dinucleotides whose DNA methylation could not be determined. Two fragments of ~300 bp and ~400 bp were amplified and sequenced. The percentage of methylated CpGs is calculated independently for each fragment.

In order to determine if the decreased expression of ELA2 in lin^-^CD34^+ ^cells from CML patients is associated with methylation of the promoter region, we isolated DNA from normal and CML patient samples. The normal samples chosen were those with the highest and lowest expression of *ELA2*, and CML1 was selected because *ELA2 *transcript levels is significantly lower than in the normal samples. Sequencing of bisulfite-treated DNA from these samples shows that there is no significant difference in methylation of the *ELA2 *promoter region between normal and CML lin^-^CD34^+ ^cells (Figure 6). Comparison of CoBRA results from CML1 (low *ELA2 *mRNA), CML4 (intermediate *ELA2 *mRNA) and CML10 (very low *ELA2 *mRNA) also did not reveal a significant difference in DNA methylation levels (data not shown). These results contrast with our initial expectation, as we had found the extent of both DNA hypomethylation and histone acetylation to be associated with *ELA2 *expression in the tested leukemic cell lines (Figures 1 and 2). However, absolute quantification of the differences in *ELA2 *transcript levels between THP-1 (*ELA2*^+^) and RPM1 (*ELA2*^-^) cell lines showed that these differed by a factor of 20,000-fold (data not shown), whereas the magnitude of average differences between the normal and CML cells was only 50-fold. It is therefore possible that the variation in expression caused by CML is too subtle to allow the role of epigenetic mechanisms to be detected.

## Discussion

In most cancers, epigenetic changes, as well as genetic aberrations, contribute to an altered program of gene expression, disease progression and increased tumor heterogeneity [26-28]. Genome-wide DNA hypomethylation leads to ectopic expression of oncogenes [29,30] and a marked increase in genomic instability [31]. Specific hypermethylation of tumor suppressor genes can act as the second hit or as the primary mechanism of transcriptional shutdown [32,33]. Furthermore, transcription promoting and inhibiting modifications to histones also occur in many cancers. These widespread epigenetic changes justify the use of chromatin remodelling drugs such as DNA methyltransferase (DNMT) and histone deacetylase (HDAC) inhibitors for the reactivation of epigenetically-silenced tumor suppressor genes. As a specific example, in CML and some Ph^+ ^acute lymphoblastic leukemias, microRNA-203 (miR-203) is silenced by DNA hypermethylation [34]. This miRNA acts as a tumor suppressor by down-regulating *ABL *through binding the 3' untranslated region (3'UTR) of *ABL *mRNA thereby preventing its translation [34].

In this study we assessed the epigenetic state of two genes, *CD7 *and *ELA2 *in primitive leukemic cells isolated from CP CML patients. Both of these genes were known to show altered expression in CML cells, and we confirmed this finding for primitive lin^-^CD34^+ ^CML cells. The extent of altered expression of both genes has also been implicated as having prognostic significance. In three human leukemic cell lines, the extent of DNA methylation and histone acetylation of the *CD7 *and *ELA2 *promoters correlated with expression. However, we found a similar correlation to be limited to the *CD7 *gene in primitive CML cells. It is possible that the higher level of *ELA2 *transcript detected in some of the CML samples is contributed by a small subset of the CD34^+ ^cells in which case an associated hypomethylation of the *ELA2 *gene would have been masked by the situation in the bulk of the cells. In addition, the difference in *ELA2 *transcript levels between the *ELA2*-high and *ELA2*-low expressing lin^-^CD34^+ ^cells was relatively small as compared to the much larger differences (20,000-fold) exhibited between *ELA2*^+ ^and *ELA2*^- ^cell lines. Thus differences in DNA methylation state amongst the primary cells might not be detectable.

*PRTN3 *(also known as *PR3*) is located ~4 kb upstream of *ELA2 *on human chromosome 19 and its expression has been shown to be co-regulated with *ELA2*. Our results confirm this co-regulation as well as indicating that, at least in our patient cohort, the transcriptional regulation of these two genes is not uncoupled in CP CML patients (Figure 3B and 3C). There is some correlation between DNA methylation of the upstream and intragenic regions of *PRTN3 *and its expression during granulopoeisis [35]. However, there is little evidence for a correlation between the amount of DNA methylation and relative abundance of *PRTN3 *transcripts in terminally differentiated granulocytes [35]. Therefore, it is plausible that differential DNA methylation, at least at the promoter region, is not the primary means of transcriptional control and transcript quantity modulation for *PRTN3 *and *ELA2*. Our results highlight the importance of comparing data from cell lines with primary patient samples for a more physiologically relevant interpretation.

A region located in intron 2 of the *PRTN3 *gene was shown to have enhancer activity for the *ELA2 *gene [36]. This enhancer element is located within a CpG island (UCSC genome browser) that stretches from exon 2 to intron 3 of *PRTN3 *and might have differential DNA methylation during granulopoeisis as well as in CML. There are two internal (intragenic) CpG islands within the *ELA2 *gene. The most 5' CpG island stretches from exon 2 to exon 3 of *ELA2 *gene (UCSC genome browser). Interestingly, a recent report covering all CpG islands of human genome shows differential methylation of this region in the primary tissue tested with the CpG island showing hypomethylation in one of two peripheral blood lymphocyte samples [37]. This region represents a potential differentially methylated region with possible regulatory roles in the transcription of *ELA2*.

Our results show that the level of *CD7 *transcript is higher in CML lin^-^CD34^+ ^cells than in their normal counterparts (Figure 3A). This finding is in accord with previous studies showing a higher proportion of CD34^+ ^cells co-expressing CD7 in CML patients versus normal individuals [38-40]. It has also been shown that chromosomal anomalies in addition to the Ph chromosome are more commonly detected in the CD34^+^CD7^+ ^subset of CML cells than in the CD34^+^CD7^- ^Ph^+ ^cells [40]. We also show that the expression of CD7 is linked to the DNA methylation of its promoter region in both primary samples and cell lines. The hypomethylation of the *CD7 *promoter region in CD34^+^CD7^+ ^cells compared to CD34^+^CD7^- ^cells of the CML-1 and CML-10 patient samples (Figure 5) denotes a likely physiological role for DNA methylation in the transcriptional repression of this gene. Whether the expression of CD7 is due to specific demethylation of its promoter or just a by-product of the genome-wide DNA hypo-methylation is unknown. However, it seems that, at least in a portion of the CD7^+ ^cells, only one allele is hypomethylated.

## Conclusions

In this study, we have shown a link between epigenetic modifications and CD7 expression in CML. There is great variability in the survival outcome of CP CML patients despite no additional detectable chromosomal abnormalities beyond the *BCR-ABL *translocation [13,41]. Changes in the epigenetic maintenance of genes such as *CD7 *could be an earlier indicator of disease progression. DNA methylation analysis can be performed reliably on as little as a few hundred cells, allowing analysis of rare cell populations. Since CD7 is an early prognostic marker detected at the CP stage, changes in its DNA methylation level, in conjunction to transcript levels, offer a potentially useful predictor of early-stage poor-prognosis CML. In light of the revived use of chromatin remodeling drugs in clinical studies, especially the various generations of DNA methyltransferase inhibitors, we believe that the state of DNA methylation of prognostic genes should be scrutinized more closely.

## Competing interests

The authors declare that they have no competing interests.

## Authors' contributions

SLR designed and performed experiments as well as data analysis; YZ performed experiments and data analysis; XJ contributed patient information; CJE provided patient samples; DLM provided research reagents and performed data analysis; AR performed experiments, data analysis and wrote the manuscript. All authors read and approved the final manuscript.

## Supplementary Material

Additional file 1 ***CD7 *and *ELA2 *expression in human leukemia cell lines**. Transcription of *ELA2 *and *PRTN3 *(A) and *CD7 *(B) is assayed with RT-PCR. GAPDH is used as endogenous control. Click here for file

Additional file 2 **DNA methylation of *CD7 *and *ELA2 *in the CML cell line K562**. CoBRA for *ELA2 *(A) and *CD*7 (B). Enzymes used are indicated as follows; U = uncut, H = *Hin*fI, R = *Rsa*, T = *Taq*Iα and M = *Mbo*I. Position of the uncut band is indicated by an arrow. Click here for file

Additional file 3 **DNA methylation analysis of *CD7 *in non-lineage-depleted CD34+ cells of CML samples**. CD34-expressing cells were FACS sorted from Ficoll-Hypaque density gradient processed CML1 and CML3 without lineage depletion and analyzed for DNA methylation status of CD7. Open and black fill circles indicate unmethylated and methylated CpG respectively. Click here for file

Additional file 4 **Significantly less *CD7 *promoter methylation in CD7 expressing cells from non-lineage-depleted CML3**. CD34-expressing cells were FACS sorted for expression of CD7 from Ficoll-Hypaque density gradient processed CML3 without lineage depletion and analyzed for DNA methylation status of CD7. Open and black fill circles indicate unmethylated and methylated CpG respectively. Click here for file
