# Supplementary figures and images for: Expression of the leukemic prognostic marker CD7 is linked to epigenetic modifications in chronic myeloid leukemia
Source: Mol Cancer. 2010 Feb 22;9:41. doi: 10.1186/1476-4598-9-41 (PMC2843654; doi:10.1186/1476-4598-9-41)

**A**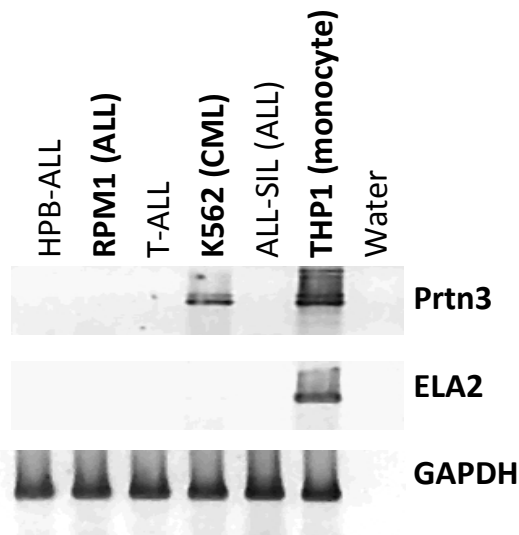**B**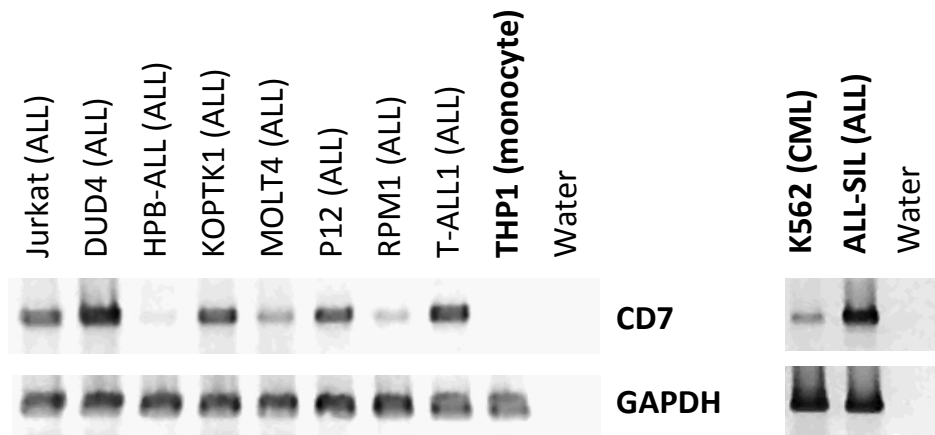

Supplement: Additional file 1 — CD7 and ELA2 expression in human leukemia cell lines. Transcription of ELA2 and PRTN3 (A) and CD7 (B) is assayed with RT-PCR. GAPDH is used as endogenous control. [file 1476-4598-9-41-S1.PDF]

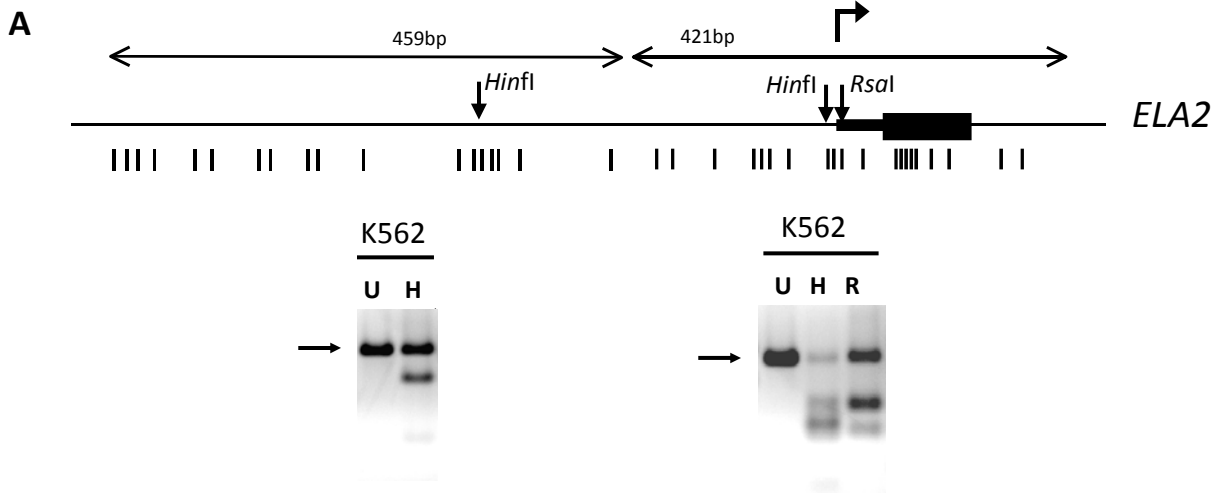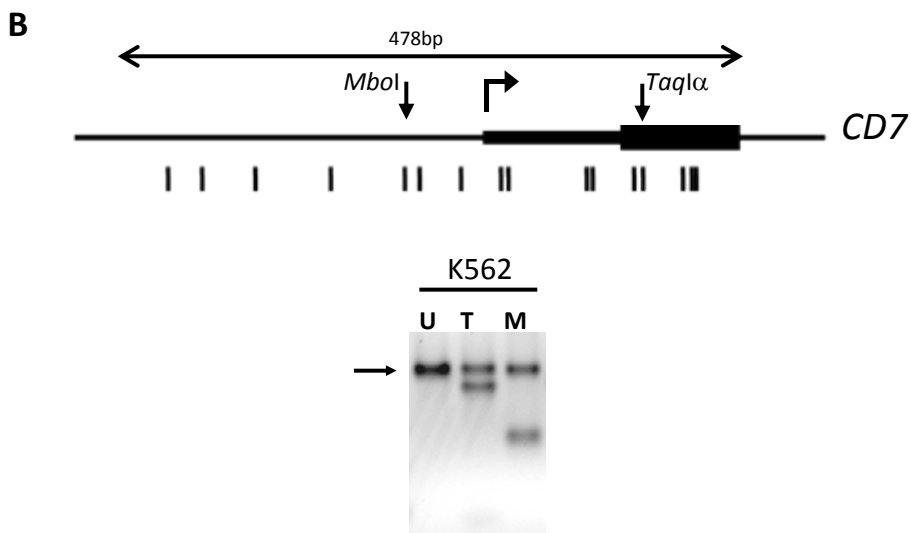

Supplement: Additional file 2 — DNA methylation of CD7 and ELA2 in the CML cell line K562. CoBRA for ELA2 (A) and CD7 (B). Enzymes used are indicated as follows; U = uncut, H = HinfI, R = Rsa, T = TaqIα and M = MboI. Position of the uncut band is indicated by an arrow. [file 1476-4598-9-41-S2.PDF]

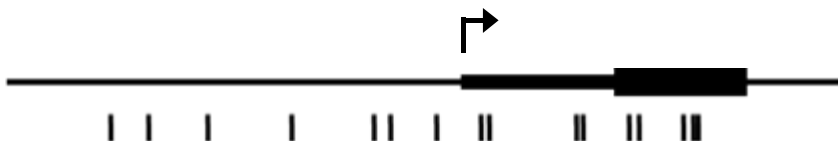

*CD7*

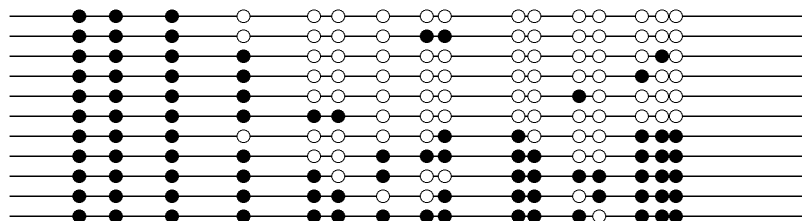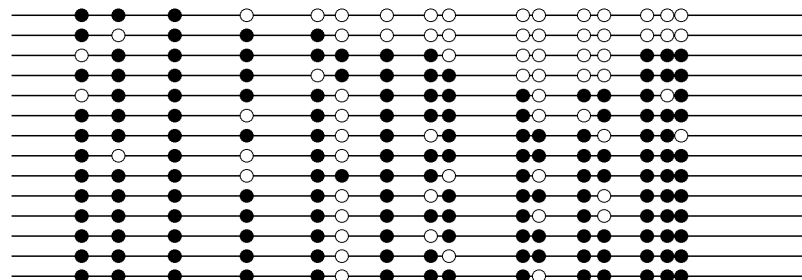

Supplement: Additional file 3 — DNA methylation analysis of CD7 in non-lineage-depleted CD34+ cells of CML samples. CD34-expressing cells were FACS sorted from Ficoll-Hypaque density gradient processed CML1 and CML3 without lineage depletion and analyzed for DNA methylation status of CD7. Open and black fill circles indicate unmethylated and methylated CpG respectively. [file 1476-4598-9-41-S3.PDF]

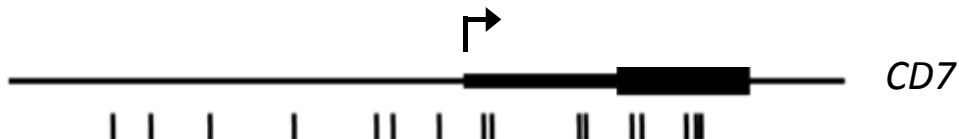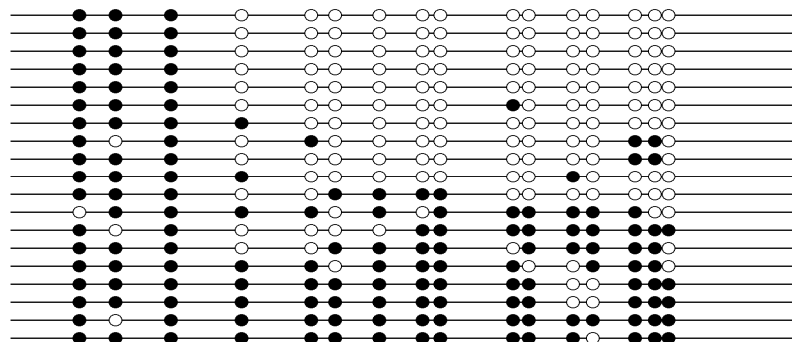

CML3 (CD34<sup>+</sup>CD7<sup>+</sup>)  
149/304 = 49% methylated

P < 0.0001

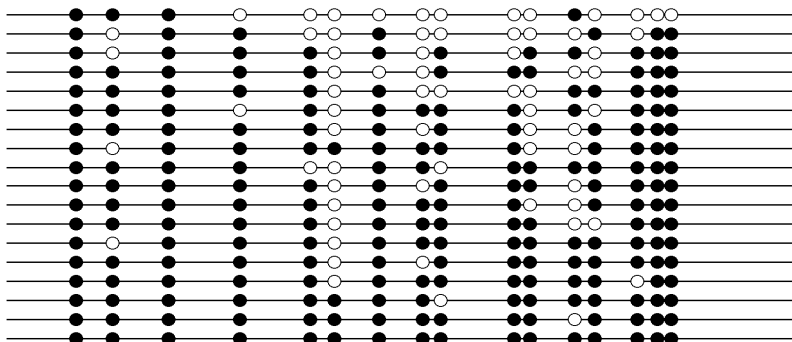

CML3 (CD34<sup>+</sup>CD7<sup>-</sup>)  
221/288 = 77% methylated

Supplement: Additional file 4 — Significantly less CD7 promoter methylation in CD7 expressing cells from non-lineage-depleted CML3. CD34-expressing cells were FACS sorted for expression of CD7 from Ficoll-Hypaque density gradient processed CML3 without lineage depletion and analyzed for DNA methylation status of CD7. Open and black fill circles indicate unmethylated and methylated CpG respectively. [file 1476-4598-9-41-S4.PDF]
